# Cryptographic Algorithm Using Newton-Raphson Method and General Bischi-Naimzadah Duopoly System

**DOI:** 10.3390/e23010057

**Published:** 2020-12-31

**Authors:** Abdelrahman Karawia

**Affiliations:** Mathematics Department, Faculty of Science Mansoura University, Mansoura 35516, Egypt; abibka@mans.edu.eg

**Keywords:** Newton-Raphson’s method, chaos, image encryption/decryption, security analysis

## Abstract

Image encryption is an excellent method for the protection of image content. Most authors used the permutation-substitution model to encrypt/decrypt the image. Chaos-based image encryption methods are used in this model to shuffle the rows/columns and change the pixel values. In parallel, authors proposed permutation using non-chaotic methods and have displayed good results in comparison to chaos-based methods. In the current article, a new image encryption algorithm is designed using combination of Newton-Raphson’s method (non-chaotic) and general Bischi-Naimzadah duopoly system as a hyperchaotic two-dimensional map. The plain image is first shuffled by using Newton-Raphson’s method. Next, a secret matrix with the same size of the plain image is created using general Bischi-Naimzadah duopoly system. Finally, the XOR between the secret matrix and the shuffled image is calculated and then the cipher image is obtained. Several security experiments are executed to measure the efficiency of the proposed algorithm, such as key space analysis, correlation coefficients analysis, histogram analysis, entropy analysis, differential attacks analysis, key sensitivity analysis, robustness analysis, chosen plaintext attack analysis, computational analysis, and NIST statistical Tests. Compared to many recent algorithms, the proposed algorithm has good security efficiency.

## 1. Introduction

Digital images play a critical role in the world today. Digital images make up 70% of the transmitted data via the Internet [1]. They often contain sensitive and valuable information which requires protection against unauthorised access in various applications such as military images, medical images and Satellite images. Therefore, researchers have been designing methods to protect digital images from piracy while they are transferred from one place to another such as encryption algorithms via chaos [2,3,4,5,6], DNA coding [7], and wavelets [8]. Also S-boxes play an excellent role in confirming the resistance of block ciphers against cryptanalysis [9]. In Reference [10], the authors presented an efficient algorithm based on a class of Mordell elliptic curves to generate S-boxes. One of the most stable and powerful public key cryptosystems has been proven to be the Elliptic Curve Cryptography, which is popular for its high performance. But improving protection by increasing the duration of the key is inefficient [11,12].

Among the many ways of image cryptography, the image cryptography based on chaotic map will selected over the past two decades. This is because the chaotic mappings have necessary proprieties such as high sensitivity to the initial conditions and the parameters, nonlinearity, non-periodicity, and pseudorandomness [13,14,15,16,17]. Numerous researchers have presented image cryptography algorithms via chaotic maps. Some of these algorithms have limited key space, weak keys, vulnerability to chosen plaintext/ciphertext attacks [18,19,20]. Almost all the authors used the permutation-substitution (confusion-diffusion) model to encrypt/decrypt the image. There are different permutation methods, from performing a shuffling to rows/columns to performing more complicated iterative processes. For example, in Reference [3], the authors proposed rows/columns shuffling algorithm using the logistic map to get permutation. Karawia in Reference [6] suggested an image encryption algorithm using Fisher-Yates shuffling to obtain permutation while Shakiba in Reference [21] performed cyclic shifts to the rows/columns via Chebyshev mapping to achieve permutation. Xiao et al. in Reference [22] used switch control mechanism to perform permutation for rows and columns of plain image. For substitution, majority of the authors applied the XOR processes [3,4,5,6,21,23], or addition modular 256 during the substitution stage of encryption [24]. There are many maps (chaotic and hyperchaotic) utilized to design encryption algorithms, for example, 1D chaotic map in Reference [25], 2D generalized Arnold map in Reference [26], 3D Cat chaotic map in Reference [27,28], and 4D chaotic map in Reference [29].

Many of the known chaotic image encryption algorithms are resistanceless for chosen plaintext attacks(CPA). These image encryption algorithms are broken by Li et al., algorithm [30], such as References [25,31,32]. To avoid this, the image encryption algorithm must be dependent on the plain image and randomized [21,33]. Based on the dimension of the chaotic map, most of 1D-chaotic maps have simple forms and simple chaotic orbits and can be guessed. So image encryption based on 1D chaotic maps are low secure [19,34]. On the contrary, the hyperchaotic maps have more complicated form and complicated chaotic performance which make expectation of their chaotic orbits is difficult [35].

In the current article, we design an image encryption algorithm that uses Newton-Raphson’s method, to shuffle the rows/columns of the plain image, and the general Bischi-Naimzadah duopoly system, to diffuse the pixels of the shuffled image. The general Bischi-Naimzada is selected to solve three essential problems: (i) the randomness of the chaotic sequences, (ii) the space of the secret key, and (iii) improving the security compared with the algorithms in literature. The chaotic sequence generated from it is extremely random. Also, it has eight parameters and two initial values and thus increasing the secret key space for the image encryption algorithm. In this algorithm, the key mixing proportion factor *K* is utilized to generate the secret key [36]. So, the proposed algorithm depends on the plain image and it can provide CPA-security. For more details about chaos based image encryption techniques, see Reference [37].

The main contributions of the current article are: (i) using a 2D chaotic map (the general Bischi-Naimzadah duopoly system) with a large positive Lyapunov exponent, wide and uniform distribution, (ii) Performing rows/columns shuffle for the plain image using pseudo-random sequence generation based on Newton-Raphson’s method, (iii) performing pixel diffusion to the shuffled image, and (iv) offering CPA-security for our algorithm.

This article is prepared as follows. In Section 2, the general Bischi-Naimzadah duopoly system is presented. The proposed algorithm is introduced in Section 3. In Section 4, security experimental results and comparative analyses are given. Finally, conclusions are mentioned in Section 5.

## 2. General Bischi-Naimzadah Duopoly System (GBNDS)

The image encryption needs a sequences of random numbers to generate a good secret image. The current paper takes advantage of the effectiveness of the general Bischi-Naimzadah duopoly system to generate pseudorandom numbers. The general Bischi-Naimzada game is a market vying between two companies based on sales constraints with the aim of maximising profits. The general Bischi-Naimzadah duopoly system is mathematically defined as [38]:

(1) $$\begin{array}{c} q_{1}\left(t+1\right)=q_{1}\left(t\right)+\nu_{1}q_{1}\left(t\right)\left[\left(1-\mu_{1}\right)\left(a-2bq_{1}\left(t\right)-bq_{2}\left(t\right)\right)-c_{1}\right]\\ q_{2}\left(t+1\right)=q_{2}\left(t\right)+\nu_{2}q_{2}\left(t\right)\left[\left(1-\mu_{2}\right)\left(a-2bq_{2}\left(t\right)-bq_{1}\left(t\right)\right)-c_{2}\right], \end{array}$$

where

$q_{i}$: the output of company i=1,2,

a>0: constant price,

b>0: the market price slope,

$c_{i}$: the marginal cost, i=1,2,

$\mu_{i}>0$: associated with the sales constraint, i=1,2,

$\nu_{i}>0$: the adjustment speed of company i=1,2.

**Figure 1:**
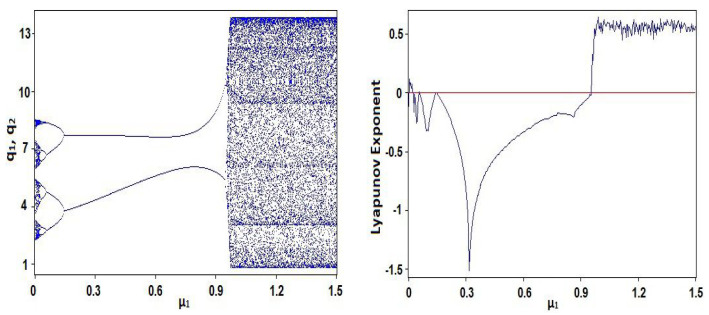
(**left**) Bifurcation diagram of system (1) regarding $\mu_{1}$, (**right**) Lyapunov exponent of system (1) regarding $\mu_{1}$.

**Figure 2:**
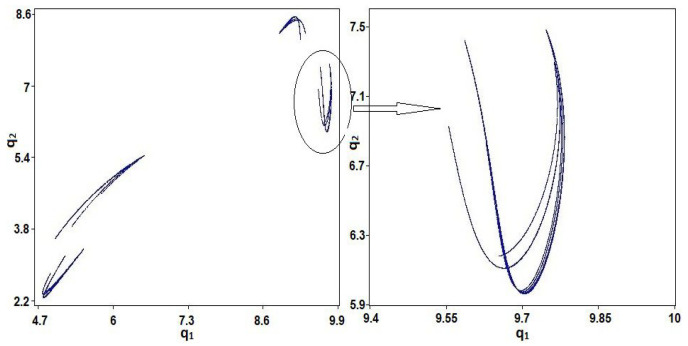
Phase diagram of system (1) for $a=11.25,b=0.5,c_{1}=0.20,c_{2}=0.30,\mu_{1}=0.002$, $\mu_{2}=0.60$, $\nu_{1}=0.20,\nu_{2}=0.70,q_{10}=0.10$, and $q_{20}=0.20$.

**Figure 3:**
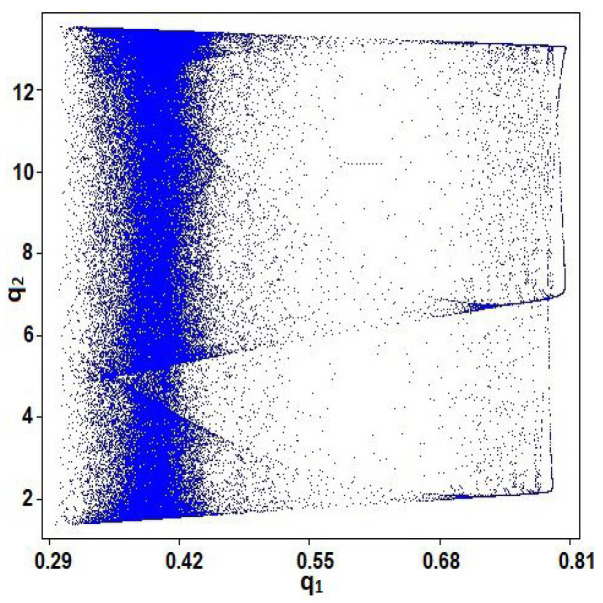
Phase diagram of system (1) for $a=11.25,b=0.5,c_{1}=0.20,c_{2}=0.30,\mu_{1}=0.97$, $\mu_{2}=0.60$, $\nu_{1}=0.20,\nu_{2}=0.70,q_{10}=0.10$, and $q_{20}=0.20$.

The chaotic behavior of system (1) is observed by the values of the parameters: a=11.25, $b=0.5,c_{1}=0.20,c_{2}=0.30,\mu_{1}=0.002,\mu_{2}=0.60,\nu_{1}=0.20,\nu_{2}=0.70$ and initial values $q_{10}=0.10,q_{20}=0.20$. Figure 1a displays the bifurcation diagram of system (1) regarding the parameter $\mu_{1}$. Lyapunov exponent of system (1) regarding the parameter $\mu_{1}$ is shown in Figure 1b. Figure 2 displays phase diagram of system (1). It presents four unconnected chaotic areas. Whereas the phase diagram of system (1) at $\mu_{1}=0.97$ is given in Figure 3 and it presents a chaotic attractor. The main advantages of the proposed coding scheme compared to other systems in the literature is that the chaotic coding sequence extracted from the **GBNDS** is extremely random. This is because this it contains many chaotic regions for different values of the parameters. The proposed system also shows a great positive feature, which is the emergence of a very wide range of chaos and complex dynamics with the parameter $\mu_{1}$ in which the system (1) shows very complex chaotic behavior [38]. Moreover, incorporating the effects of sales constraints into the form has the advantage of increasing the number of parameters in the form and thus expanding the secret key space for the cryptography process. Also, a stable coexistence of multiple chaotic attractions is observed in this case [38].

## 3. The Proposed Algorithm

The proposed method applies confusion-diffusion model to encrypt the plain image. Sequences are generated by the points, calculated by Newton-Raphson’s method, on a polynomial function. Based on these sequences, the rows/columns of the plain image are shuffled (confusion phase). By applying XOR between the shuffled image and the generated values of the chaotic system (1), the diffusion stage modifies the pixel values. In the current section, the key generation, rows/columns Shuffling, and an image encryption/decryption algorithms are presented.

### 3.1. The Key Generation

Suppose that $O=(o_{ij}),\;i=1,2,\ldots ,M,$ and j=1,2,…,N, is the plain image. The secret key is generated by using the key mixing proportion factor *K* as follows [36]:

(2) $$K_{s}=\frac{1}{256}mod\left(\sum_{i=[\frac{(s-1)M}{2}]+1}^{\left[\frac{sM}{2}\right]}\sum_{j=1}^{N}o_{ij},256\right),\;s=1,2,$$

and, the key values $\zeta_{s}$ is changed via the following formula:

(3) $$\zeta_{s}\leftarrow \frac{\left(\zeta_{s}+K_{s}\right)}{2},\;s=1,2,$$

where [x] denoted to the nearest integer and $\zeta_{s}$ denoted to $q_{s0},\;\;\;s=1,2.$

The key space of the proposed algorithm consists of the polynomial function with degree *k* and limits of [α,β] for the Newton-Raphson’s method, two initial values and eight parameters for GBNDS. Then, for the confusion phase, select two values, α,β, and one polynomial function based on Newton-Raphson’s method, and for diffusion phase, two initial values, $q_{10},q_{20}$, and eight parameters $a,b,c_{1},c_{2},\mu_{1},\mu_{2},\nu_{1},\nu_{2}$ for the System (1).

### 3.2. Rows/Columns Shuffling (Confusion Phase)

In this section, we design a technique for generating a random permutation of the integers {1,2,…,n}. Then, we shuffle the rows/columns of the plain image via the random permutation sequences.

Suppose a polynomial function of degree *s*, $p\left(x\right)=\sum_{i=1}^{s}a_{i}x^{i},$ where $a_{s}\neq 0$ and s>1, is defined on the interval [α,β]. Take $x_{0}=\left(\alpha +\beta \right)/2$ and Newton-Raphson’s method generates the sequence ${\left\{x_{i}\right\}}_{i=0}^{\infty }$ by the following formula:

(4) $$x_{i}=x_{i-1}-\frac{p(x_{i-1})}{p^{\prime }\left(x_{i-1}\right)},\;p^{\prime }\left(x_{i-1}\right)\neq 0\;\forall \;i=1,2,3,\ldots$$

Suppose, the Newton-Raphson’s method generates the points $\left\{x_{1},x_{2},x_{3},\ldots ,x_{n}\right\}$. To get more randomness, instead of $p(x_{i})$, the sequence is defined as the fraction part of $p(x_{i})$. This sequence depends on the polynomial p(x) and the interval [α,β].

The standard NIST SP800-22 test is used to assess the efficiency of the pseudorandom number generator(PRNG) of Newton-Raphson’s method, and Table 1 gives the test results. In Table 1, the random number generator has passed all the tests. So, it has a good randomness.

Algorithm 1 is proposed to generate a random permutation of the integers {1,2,…,n} based on Newton-Raphson’s method as follows:
**Algorithm 1** Random-Permutation algorithm **Input:** Size of random numbers, *n*, the polynomial p(x), α, and β. **Output:**
*S*, the random permutation of the integers {1,2,…,n}. **Step 1:** Set S=1, $x_{0}=\left(\alpha +\beta \right)/2$, $x=p\left(x_{0}\right)-fix\left(p\left(x_{0}\right)\right)$ **Step 2:** For i=2 to *n*, compute              S=[Si]              k=ceil(i∗x)              S([ki])=S([ik])              $x_{1}=x_{0}-p\left(x_{0}\right)/p^{\prime }\left(x_{0}\right)$              $x=p\left(x_{1}\right)-fix\left(p\left(x_{1}\right)\right)$              $x_{0}=x_{1}$            End For **Step 3:**
*S*

Suppose the size of the plain image is M×N. Algorithm 2 is designed to shuffle the plain image based on the random permutation sequences of Algorithm 1. It may be processed as in Algorithm 2.

### 3.3. Diffusion Phase

The system (1) is utilized to generate a chaotic sequence of size M×N. Then, reshape it to be of size 1×MN, $Q=\{q_{1},q_{2},\ldots ,q_{MN}\}$. The sequence *Q* is modified using the following formula:

(5) $$q_{i}=mod\left(ceil\left(q_{i}\times 10^{14}\right),256\right),i=1,2,\ldots ,MN.$$

**Algorithm 2** Row/Columns shuffling algorithm **Input:** The plain image, *O*, the polynomial p(x), $\alpha_{1},\alpha_{2},\beta_{1}$, and $\beta_{2}$. **Output:**
*H*, the shuffled image. **Step 1:** Set [M,N]=size(O) **Step 2:** Use Algorithm 1, with polynomial p(x), and interval $\left[\alpha_{1},\beta_{1}\right]$, to generate a random           permutation of size *M* for shuffling the rows, say $S_{Rows}$. **Step 3:** Use Algorithm 1, with polynomial p(x), and interval $\left[\alpha_{2},\beta_{2}\right]$, to generate a random           permutation of size *N* for shuffling the columns, say $S_{Columns}$. **Step 4:** For i=1 to *M*, compute                  For j=1 to *N*, compute                       $H\left(i,j\right)=O(S_{Rows}\left(i\right),S_{Columns}\left(j\right))$                   End For j            End For i **Step 5:**
*H*, the shuffled image.


Moreover, the shuffled image *H* is reshaped to be of size 1×MN, $H=\{h_{1},h_{2},\ldots ,h_{MN}\}$. Finally, XOR is applied between each pixel in *H* and corresponding chaotic value of *X*, D=XOR(H,X) (diffusion phase). The algorithm of diffusion phase may be processed as follows:
**Algorithm 3** Diffusion algorithm **Input:** The shuffled image, *H*, $q_{10},q_{20},a,b,c_{1},c_{2},\mu_{1},\mu_{2},\nu_{1}$, and $\nu_{2}$. **Output:**
*D*, the diffusion vector. **Step 1:** Reshape *H*, $H=\{h_{1},h_{2},\ldots ,h_{MN}\}$.  **Step 2:** Covert *H* to binary, $H_{b}$. **Step 3:** Set $q_{1}\left(0\right)=q_{10},q_{2}\left(0\right)=q_{20}$. **Step 4:** Perform initial iterations,          For t=0 to 999                  $q_{1}\left(t+1\right)=q_{1}\left(t\right)+\nu_{1}q_{1}\left(t\right)\left[\left(1-\mu_{1}\right)\left(a-2bq_{1}\left(t\right)-bq_{2}\left(t\right)\right)-c_{1}\right]$
                  $q_{2}\left(t+1\right)=q_{2}\left(t\right)+\nu_{2}q_{2}\left(t\right)\left[\left(1-\mu_{2}\right)\left(a-2bq_{2}\left(t\right)-bq_{1}\left(t\right)\right)-c_{2}\right]$        End For **Step 5:** Set $q_{1}\left(0\right)=q_{1}\left(1000\right),q_{2}\left(0\right)=q_{2}\left(1000\right)$. **Step 6:** For t=0 to MN−1                  $q_{1}\left(t+1\right)=q_{1}\left(t\right)+\nu_{1}q_{1}\left(t\right)\left[\left(1-\mu_{1}\right)\left(a-1\left(t\right)-bq_{2}\left(t\right)\right)-c_{1}\right]$
                  $q_{2}\left(t+1\right)=q_{2}\left(t\right)+\nu_{2}q_{2}\left(t\right)\left[\left(1-\mu_{2}\right)\left(a-2bq_{2}\left(t\right)-bq_{1}\left(t\right)\right)-c_{2}\right]$                  $q\left(t+1\right)=(q_{1}\left(t+1\right)+q_{2}\left(t+1\right))/2$        End For **Step 7:** Preprocess the values of Q={q(1),q(2),…,q(MN)} as follows:          $q\left(t\right)=mod(ceil\left(q\left(t\right)*10^{14}\right),256),t=1,2,\ldots ,MN$. **Step 8:** Covert *Q* to binary, $Q_{b}$. **Step 9:** Perform XOR between $H_{b}$ and $Q_{b}$, say $D=XOR(H_{b},Q_{b})$.


### 3.4. The Encryption/Decryption Algorithm

The encrypted image is produced from Algorithm 3 by reshape diffusion vector *D* to be of size M×N, say *E*. The whole image encryption algorithm may be processed as in the Algorithm 4.

The Algorithm 4 (Image Encryption based on General Bischi-Naimzadah Duopoly System) will be referred to as **IEGBNDS** algorithm. Indeed, **IEGBNDS** algorithm can be applied to encrypt the color images. We can decompose color images into three grayscale images of red, green and blue colors (R, G, B components). After that we can encrypt them into their corresponding cipher images by applying the proposed algorithm. Then by re-joining the three cipher images of the R, G, B components, the color cipher image can be obtained.
**Algorithm 4** Image encryption algorithm **Input:** The plain image, *O*, the polynomial p(x), α, β, $q_{10},q_{20},a,b,c_{1},c_{2},\mu_{1},\mu_{2},$
$\nu_{1}$, and $\nu_{2}$. **Output:**
*E*, the encrypted image. **Step 1:** Read the plain image, *O*. **Step 2:** Generate the secret key by using the key mixing proportion factor. **Step 3:** Call Algorithm 2 to get the shuffled image *H*. **Step 4:** Call Algorithm 3 to get the diffusion vector *D*. **Step 5:** Covert *D* to decimal, say $D_{d}$. **Step 6:** Change the dimension of $D_{d}$ to M×N, say *E*. **Step 7:**
*E* is the encrypted image.


**Figure 4:**
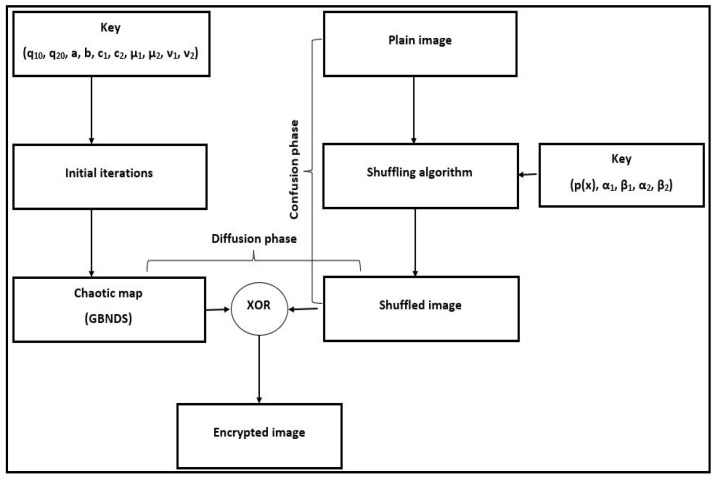
Block diagram of the proposed algorithm.

The decryption algorithm is the inverse steps of **IEGBNDS** algorithm. Figure 4 displays the block diagram of **IEGBNDS** algorithm.

## 4. Experimental Results

The **IEGBNDS** algorithm has been applied to several 512×512 pixel gray-scale images and very promising results have been accomplished. All codes are accomplished on a Windows 10 Laptop with Intel(R) Core(TM) i7 2.40 GHz, CPU with 12 GB RAM using MATLAB R2016b.

### 4.1. Key Space Analysis

The key space must be large enough to hold out against brute-force attack. It must be above the value $2^{100}$[39]. The key space of the **IEGBNDS** algorithm consists of the polynomial function with degree *k* and limits of [α,β] for the Newton-Raphson’s method, two initial values and eight parameters for GBNDS. If the accuracy $10^{-14}$ has been used then it will be equal to $10^{14(k+1)}+10^{168}\left(>>2^{100}\right)$. Table 2 gives the key space of the **IEGBNDS** algorithm compared to some recent algorithms in literature.

### 4.2. Histogram Analysis

**Figure 5:**
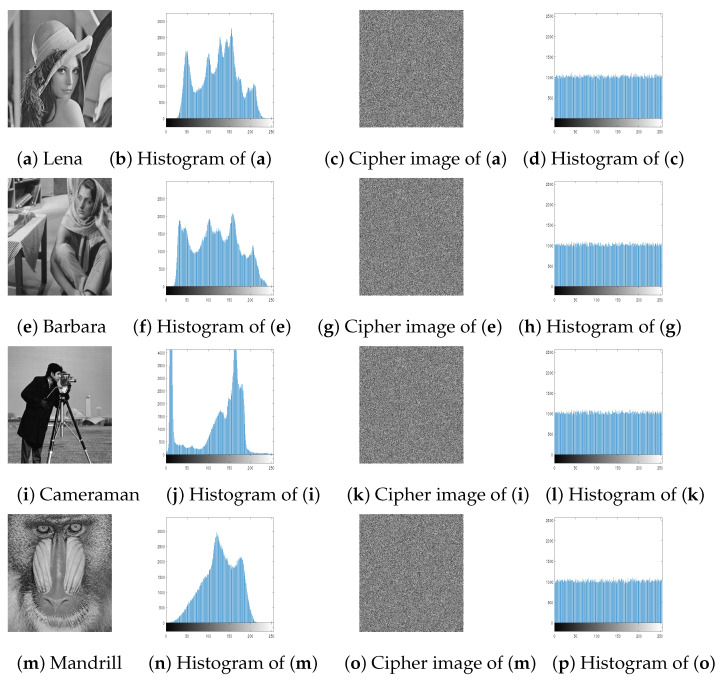
Plain images, cipher images and their corresponding histograms.

**Figure 6:**
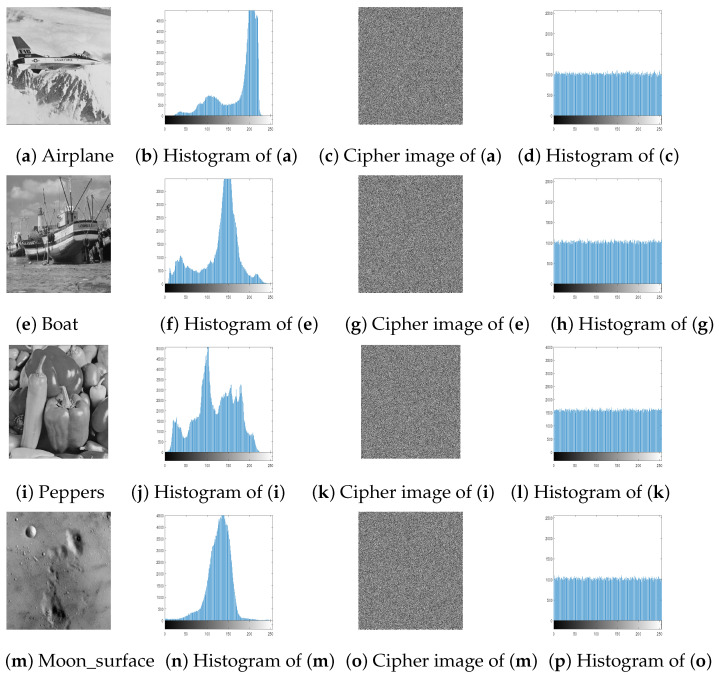
Plain images, cipher images and their corresponding histograms.

In a good encryption algorithms, the distribution of the pixel intensity values within a cipher image should be as similar to the uniform distribution as possible. Figure 5 and Figure 6 show that the histograms of the cipher image is very similar to the uniform distribution. As the $\chi^{2}$ statistical test is used to measure the nearness of produced histograms to the uniform histogram. The statistical $\chi^{2}$-value is evaluated by [6]:

(6) $$\chi^{2}=\sum_{i=1}^{256}\frac{{\left(E_{i}-e_{i}\right)}^{2}}{e_{i}},$$

where the length of all possible values in an image is 256, $E_{i}$ is the observed event frequencies of i−1 and $e_{i}$ is the expected event frequencies of i−1, i=1,2,…,256. By evaluating the $\chi^{2}$-value with the level of significance α=0.05, we got $\chi_{0.05}\left(255\right)=293.25$. So, Both distributions are nearly equal if $\chi^{2}\left(255\right)<293.25$. Table 3 shows that all tested images are smaller than 293.25. Therefore, the cipher images histograms are close to the uniform distributions. In other words, an attacker cannot retrieve any valuable information from them.

#### Histogram Statistics

The variance and standard deviation are dispersion metrics applied in graphic histograms to help the effects of visual inspection. They calculate how often the elements of a dataset differ across the average with respect to each other. The same average value (mean) can be in two datasets, but the differences may be dramatically different. If the histogram has the lower variance then it has the more uniform of the graphic histogram, which is calculated by the following formula:

(7) $$V=\frac{1}{256}\sum_{i=1}^{256}{\left(\theta_{i}-\bar{\theta }\right)}^{2},$$

where

(8) $$\bar{\theta }=\frac{M\times N}{256},$$

$\theta_{i}$ is the frequency for each pixel’s value from 0−255 of the histogram, i=1,2,…,256, $\bar{\theta }$ is the histogram mean.

The standard deviation helps us to know the arithmetic average of the dataset’s variations relative to the mean. It is calculated as follows:

(9) $$S=\sqrt{V},$$

where *V* is the histogram variance.

Table 4 presents the histogram statistics for the plain and cipher images of the tested images for the **IEGBNDS** algorithm and the encryption algorithm in Reference [41].

### 4.3. Entropy Analysis

Information entropy [7] is utilized to detect the randomness of the cipher image. It is computed as follows:

(10) $$H=\sum_{i=0}^{255}P_{i}log_{2}\left(\frac{1}{P_{i}}\right),$$

where $P_{i}$ is the probability associated with gray level *i*. The largest value of the entropy reflects the randomness of the encrypted image. The maximum value of the entropy in our case is 8. Table 5 gives the information entropy for the plain and cipher images of the tested images. All values of entropy based on our algorithm are close to 8. In addition, the **IEGBNDS** algorithm gives average better than most averages of the listed recent algorithms. Based on the results of entropy, the **IEGBNDS** algorithm has reasonable protection.

### 4.4. Correlation Coefficients Analysis

**Figure 7:**
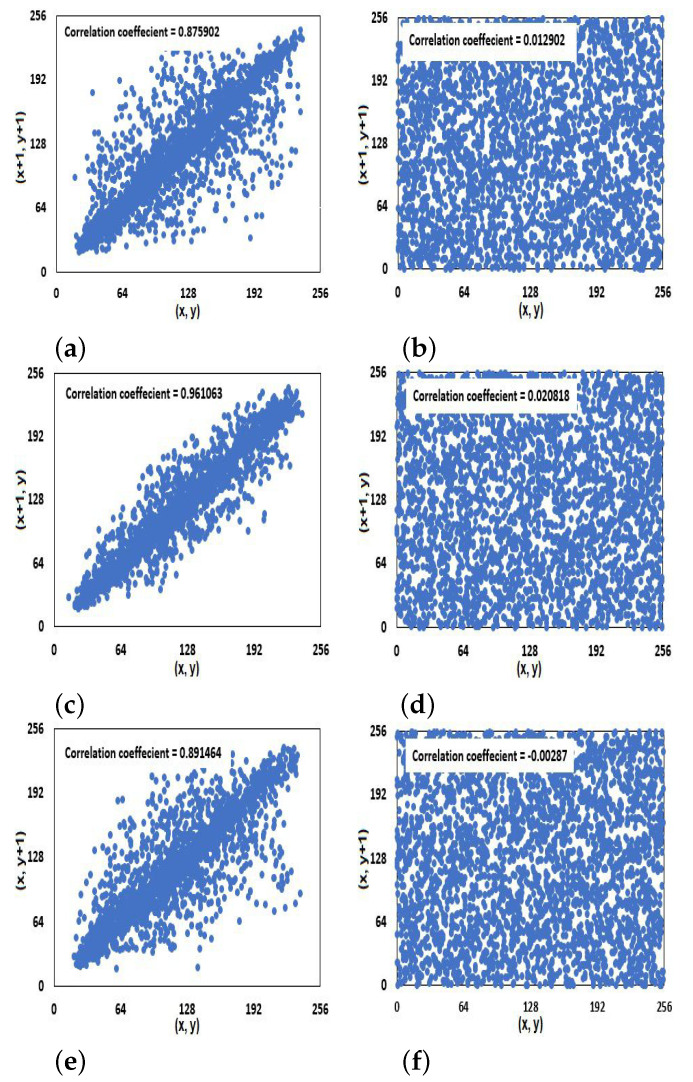
Distribution of adjacent pixels in the plain image (**a**,**c**,**e**) and the cipher image (**b**,**d**,**f**) for Barbara image in the three directions, diagonal, horizontal, and vertical.

In the plain image, adjacent pixels have strong relationships. So, reducing these relationships is required to hold out against statistical attacks. The correlation coefficient between two adjacent pixels, θ and ϕ, is defined as [6]:

(11) $$r_{\theta \phi }=\frac{Cov(\theta ,\phi )}{\sqrt{(}D\left(\theta \right)D\left(\phi \right))},$$

where

(12) $$Cov\left(\theta ,\phi \right)=\frac{1}{N}\sum_{m=1}^{N}\left(\theta_{m}-E\left(\theta \right)\right)\left(\phi_{m}-E\left(\phi \right)\right),$$

(13) $$E\left(\theta \right)=\frac{1}{N}\sum_{m=1}^{N}\theta_{m},$$

and

(14) $$D\left(\theta \right)=\frac{1}{N}\sum_{m=1}^{N}{\left(\theta_{m}-E\left(\theta \right)\right)}^{2},$$

where θ and ϕ are selected randomly. 3000 pairs of adjacent pixels are chosen randomly from the plain and cipher images. Figure 7 displays the pixel intensity value’s distribution of 3000 pairs for the Barbara image and its encrypted image in the three directions, diagonal, horizontal, and vertical. The correlation coefficients of the three directions for the **IEGBNDS** algorithm compared to some recent encryption algorithms based on the average of the correlation coefficients are given in Table 6. Table 6 shows that the **IEGBNDS** algorithm outperforms all of them at least in one direction. Also all values of $r_{\theta \phi }$ for the cipher images are close to zero. So, it can protect the image information.

### 4.5. Differential Attack Analysis

The protection against differential attacks is required for any image encryption algorithm. There are two main measurements, (1) NPCR (Number of Pixel Change Rate), and (2) UACI (Unified Average Changing Intensity). These measurements evaluated the amount of differences between two images, and can be defined as [5]:

(15) $$NPCR=\frac{\sum_{m,n}D\left(m,n\right)}{M\times N}\times 100\%,$$

(16) $$UACI=\frac{1}{M\times N}\left[\sum_{m,n}\frac{\left|O(m,n)-E(m,n)\right|}{255}\right]\times 100\%,$$

where

(17) $$D\left(m,n\right)=\left\{\;\begin{array}{cc} 0 & \;\;\mathrm{if}\;O(m,n)=E(m,n),\\ 1 & \;\;\mathrm{otherwise}. \end{array}\right.$$

A single pixel of the plain image is selected randomly and it modified to 255−v, where *v* is the original intensity value of pixel. The same key is utilized to encrypt the modified image and the plain image. Then, NPCR, and UACI are calculated using the two cipher images. Table 7 shows NPCR and UACI for the tested images and compared them to some recent algorithms in literature. The **IEGBNDS** algorithm offers a good level of security. Based on the averages of NPCR and UACI, the **IEGBNDS** algorithm outperforms all of them at least in one of the two measures. So, the **IEGBNDS** algorithm can be useful against differential attacks.

### 4.6. Key Sensitivity Analysis

The sensitivity to the secret key is one of the important features of an excellent encryption algorithm. During the restoring plain image (decryption process), small changes in one of the initial values or parameter are made and we will observe the restoring image via the modified secret key. Table 8 shows the restoring images using the true secret key and the modified secret keys. The plain image cannot be restored by any of modified secret keys. Therefore, the **IEGBNDS** algorithm is highly sensitive to any changes of the secret key.

### 4.7. Robustness Analysis

In real life, noise or data loss is occurred and the **IEGBNDS** algorithm is tested against these problems. Salt&Pepper noise with different densities are added to the cipher image of lena with size 512×512. Table 9 shows the decrypted images of the noisy encrypted images. Moreover, the decryption image of the encryption image with some data loss is shown in Table 9. Based on the result of Table 9, The **IEGBNDS** algorithm can be robust against the noise and data loss attacks.

### 4.8. Chosen Plaintext Attack Analysis

The **IEGBNDS** algorithm is sensitive to the key generation, $K_{s}$, in Equation (2) and different sequences will be generated by small changes in the plain image. So, the **IEGBNDS** algorithm can hold out against the plaintext attacks. Now, we will examine the **IEGBNDS** algorithm against the chosen plaintext attack. Suppose the attacker has the encrypted image and the running of the **IEGBNDS** algorithm for a short time. The algorithm of Reference [42] will be used to examine our algorithm against chosen plaintext attack. In this algorithm, the following notations will be used:

*P*: plain image,
*E*: encrypted image of *P*,
*D*: designed image, where $d_{mn}=0,\;m=1,2,\ldots ,M,\;n=1,2,\ldots ,N$,
$E_{D}$:  encrypted image of *D*,
$D_{E}$:  decrypted image of *E*.

**Figure 8:**
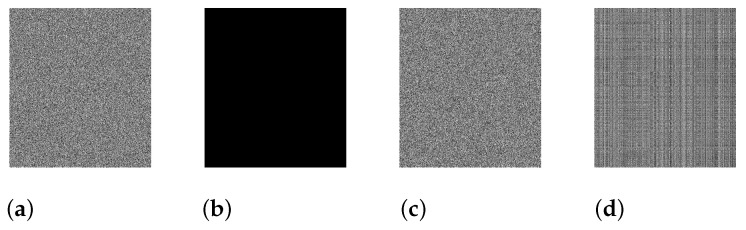
Analysis of chosen plaintext attack: (**a**) Encrypted image *E*, (**b**) designed image *D*, (**c**) encrypted image of *D*, (**d**) Decrypted image $D_{E}$.

The XOR operations between the pixels of *E* and $E_{D}$ are performed to obtain the plain image *P*. Based on the result of Figure 8, the decrypted image is totally unlike the plain image. Therefore, the **IEGBNDS** algorithm can resist chosen plaintext attack.

### 4.9. Computational Analysis

The average times of encryption and decryption algorithm for one hundred time are 34.11 ms and 30.57 ms (Tested image of size 512×512), respectively. On the other hand, for the tested image of size M×N, the encryption algorithm needs 5 MN+M+N+2000 operations. The complexity time for the decryption algorithm is equal to the complexity time of the encryption algorithm. Table 10 shows that the running time of the **IEGBNDS** algorithm is effective compared to some recent image encryption algorithms such as in Reference [40] by Shakiba and Reference [23] by Cao et al.

### 4.10. NIST Statistical Tests

NIST were established to test the randomness of generating cipher images created by encryption algorithms [43]. For the **IEGBNDS** algorithm, it is used to check the randomness of a sequence that consists of 100 cipher images of length 512×512×8=2,097,152 bits. They were generated by using different random secret keys. Table 11 presents the results for 15 tests and all of them passed these tests.

## 5. Conclusions

In this article, the **IEGBNDS** algorithm via Newton-Raphson’s method and general Bischi-Naimzadah duopoly system (**GBNDS**) has been suggested. Newton-Raphson’s method has been used for shuffling the rows/columns of the plain image. **GBNDS** has been used to producing chaotic sequences to diffusion phase of image encryption algorithm. The extracted chaotic sequences from the **GBNDS** is extremely random based on the NIST statistical tests. Many security experiments are applied to evaluate the efficiency of our algorithm. The **IEGBNDS** algorithm has a large key space ($10^{14(k+1)}+10^{168}\left(>>2^{100}\right)$, the histograms of the generated cipher images are close to the uniform distributions, all entropy values for the cipher images based on **IEGBNDS** algorithm are close to 8, all correlation coefficient values for the cipher images are close to zero. The **IEGBNDS** algorithm outperforms some recent algorithms at least in one of the two measures, highly sensitive to small changes of the secret key, can be robust against the noise and data loss attacks, and can hold out against the plaintext attacks. In comparison to several recent algorithms, the **IEGBNDS** algorithm has a small running time. NIST statistical tests for 100 cipher images by the **IEGBNDS** algorithm are performed and all tests are passed. Finally, quantum image encryption algorithm based on **GBNDS** will be designed in the future to increase the security of the current algorithm. 

## Figures and Tables

**Table 1:** NIST statistical test for PRNG-Newton-Raphson’s method.

Statistical Test	PRNG	Result
Frequency monobit test	100/100	PASS
Block frequency test	99/100	PASS
Rank test	99/100	PASS
Runs test	97/100	PASS
Longest runs test	99/100	PASS
Cumulative sums test	100/100	PASS
Discrete Fourier transform	100/100	PASS
Random excursion test	56/58	PASS
Random excursion variant test	57/58	PASS
Universal test	96/100	PASS
Approximate entropy	97/100	PASS
Linear complexity test	99/100	PASS
Serial	99/100	PASS
Non Overlapping templates test	97/100	PASS
Overlapping templates test	100/100	PASS

**Table 2:** Key space of the **IEGBNDS** algorithm compared to some recent algorithms in literature.

Algorithm	IEGBNDS Algorithm	**[** **5** **]**	**[** **23** **]**	**[** **40** **]**
Key space	$\left(10^{14(k+1)}+10^{168}\right)>2^{605}$	$10^{140}\approx 2^{466}$	>10${}^{4}\times 2^{208}$	$2^{256}$

**Table 3:** $\chi^{2}$-values of the histograms of the cipher images at $a=11.25,b=0.5,c_{1}=0.20$, $c_{2}=0.30$, $\mu_{1}=0.002,\mu_{2}=0.60,\nu_{1}=0.20,\nu_{2}=0.70,q_{10}=0.10$, and $q_{20}=0.20$.

Image	$\chi^{2}$-Value
Lena	286.52
Barbara	256.74
Cameraman	252.88
Mandrill	275.24
Airplane	279.64
Boat	260.23
Peppers	288.74
Moon_surface	249.12

**Table 4:** Histogram statistics for the **IEGBNDS** algorithm and the encryption algorithm in Reference  [41].

Image	Plain Image		Cipher Image			
			IEGBNDS		**[** **41** **]**	
	V	S	V	S	V	S
lena (256×256)	38451	196.1	396	19.9	414	20.3
lena (512×512)	633397	795.9	3171	56.3	3340	57.8

**Table 5:** Information entropy analysis of the **IEGBNDS** algorithm compared to some recent algorithms in literature.

Image	Information Entropy	
	Plain Image	Encrypted Image
Lena	7.4475	7.9992
Barbara	7.6338	7.9993
Cameraman	7.0518	7.9993
Mandrill	7.2933	7.9992
Airplane	6.6823	7.9992
Boat	7.2151	7.9993
Peppers	7.4849	7.9992
Moon_surface	6.6974	7.9993
Average	7.1883	7.99925
[5] (Average)	–	7.99867
[23] (Average)	–	7.90252
[40] (Average)	7.266297	7.999224

**Table 6:** Correlation coefficient of the cipher images based on the **IEGBNDS** algorithm compared to some recent encryption algorithms in literature.

Image	Correlation Coefficient		
	Horizontal	Vertical	Diagonal
Lena	0.0011	−0.0026	−0.0015
Barbara	−0.0006	−0.0015	−0.0010
Cameraman	−0.0035	−0.0029	−0.0015
Mandrill	−0.0002	−0.0005	−0.0026
Airplane	0.0029	−0.0020	−0.0049
Boat	−0.0034	−0.0019	0.0010
Peppers	−0.0035	0.0032	0.0017
Moon_surface	−0.0013	0.0000	0.0011
Average	0.002063	0.001825	0.001913
[5] (Average)	0.007067	0.007867	0.014567
[23] (Average)	0.001544	0.001772	0.002678
[40] (Average)	0.003134	0.006602	0.004525

**Table 7:** NPCR and UACI of the tested images using the **IEGBNDS** algorithm and the recent algorithms.

Image	NPCR (%)	UACI%
Ideal value [23]	99.6094	33.4635
Lena	99.6326	33.4584
Barbara	99.6082	33.5339
Cameraman	99.6044	33.5797
Mandrill	99.6204	33.4392
Airplane	99.5907	33.4608
Boat	99.6086	33.4599
Peppers	99.6033	33.4868
Moon_surface	99.5998	33.4590
**Average**	99.6085	33.48471
[5] (Average)	99.6067	33.4267
[23] (Average)	99.6083	33.4521
[40] (Average)	99.6060	33.4646

**Table 8:** The result of key sensitivity analysis.

Cipher	Decrypted with	Decrypted with	Decrypted with
image	true key	wrong key $q_{1}\left(1\right)\times 10^{-14}$	wrong key $q_{2}\left(1\right)\times 10^{-14}$
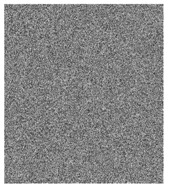	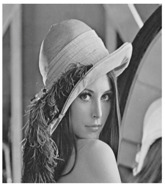	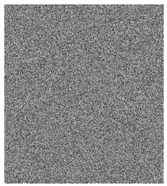	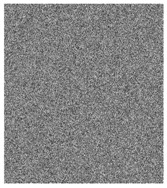
Decrypted with	Decrypted with	Decrypted with	Decrypted with
wrong key $a\times 10^{-14}$	wrong key $b\times 10^{-14}$	wrong key $\mu_{1}\times 10^{-14}$	wrong key $\nu_{1}\times 10^{-14}$
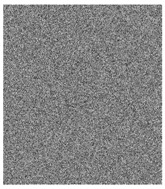	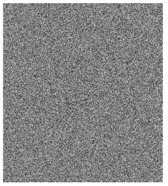	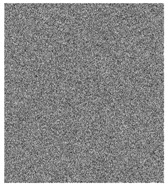	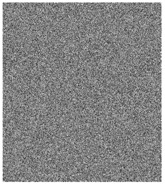

**Table 9:** Robustness analysis of the **IEGBNDS** algorithm for lena image with size 512×512.

Encrypted with	Decryption of	Encrypted with	Decryption of
salt&pepper(0.01)	previous image	salt&pepper(0.05)	previous image
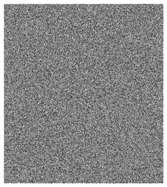	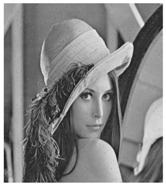	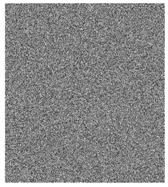	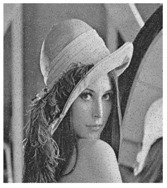
Encrypted with	Decryption of	Encrypted with	Decryption of
salt&pepper(0.1)	previous image	corp of 200×200	previous image
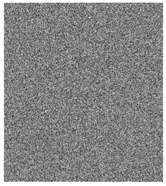	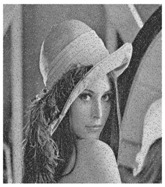	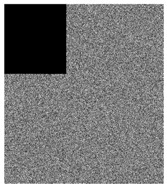	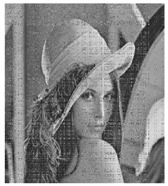

**Table 10:** Running time of the encryption for the **IEGBNDS** algorithm and the recent algorithms.

Algorithm	Image Size	Running Time (ms)
**IEGBNDS**	512×512	34.11
[40]	512×512	976±24.6
[23]	256×256	32.43

**Table 11:** NIST statistical test for 100 cipher images by the **IEGBNDS** algorithm.

Statistical Test	IEGBNDS Algorithm	Result
Frequency monobit test	100/100	PASS
Block frequency test	99/100	PASS
Rank test	99/100	PASS
Runs test	99/100	PASS
Longest runs test	100/100	PASS
Cumulative sums test	99/100	PASS
Discrete Fourier transform	98/100	PASS
Random excursion test	56/58	PASS
Random excursion variant test	57/58	PASS
Universal test	99/100	PASS
Approximate entropy	98/100	PASS
Linear complexity test	100/100	PASS
Serial	100/100	PASS
Non Overlapping templates test	99/100	PASS
Overlapping templates test	100/100	PASS

## Data Availability

Data sharing not applicable.
